# The texture of collagen in the microenvironments of Merkel cell carcinoma

**DOI:** 10.1097/MD.0000000000027925

**Published:** 2021-11-24

**Authors:** Tiago Luders Laurito, Flávia Thomé França, Gislaine Vieira-Damiani, Vitor Bianchin Pelegati, Mariana Ozello Baratti, Hernandez Faustino de Carvalho, Carlos Lenz Cesar, Aparecida Machado de Moraes, Maria Letícia Cintra, Fernanda Teixeira

**Affiliations:** a Department of Pathology, Faculty of Medical Sciences, State University of Campinas, Rua Tessália Vieira de Camargo, 126. Cidade Universitária Zeferino Vaz, Campinas, SP, Brazil; b Federal Institute of Education, Science and Technology of São Paulo, Avenida Ênio Pires de Camargo, 2971, Capivari, SP, Brazil; c National Institute of Photonics Applied to Cell Biology, Department of Quantum Electronics, Institute of Physics, State University of Campinas, Rua Sergio Buarque de Holanda, 777, SP, Brazil; d Department of Dermatology, Faculty of Medical Sciences, State University of Campinas, Rua Tessália Vieira de Camargo, 126. Cidade Universitária Zeferino Vaz, Campinas, SP, Brazil.

**Keywords:** collagen fibers, Merkel cell carcinoma, nonlinear optical microscopy, second harmonic generation, textural analysis

## Abstract

Solid tumors typically contain high levels of fibrillar collagen. The increased stromal collagen deposition usually promotes cancer progression since biochemical and biophysical cues from tumor-associated collagen fibers stimulate neoplastic cells. Few studies have investigated the relationship between Merkel cell carcinoma (MCC) and the extracellular matrix (ECM), but there are no works evaluating collagen.

This is an observational, analytical, retrospective study including 11 patients with MCC. Primary tumor-stained sections were evaluated by second harmonic generation microscopy and texture analysis.

Peritumoral texture features (area fraction, mean gray value, entropy, and contrast) showed much lower values than normal skin (*P* < .0001) revealing extensively altered structure of peritumoral collagen fibers. These differences were not significant between tumors with unfavorable and favorable known prognostic factors.

Profound changes in collagen fibers present in the stroma accompanying primary MCC may contribute to the aggressive behavior of this tumor. Our results indicate that whatever MCC histological subtype, size or anatomical location, MCC promotes the same type of ECM for its development. As an outlook, therapies using ECM macromolecules or fibroblasts (the architects of ECM remodeling) as target could be useful in the treatment of MCC.

## 1 Introduction

**Figure 1:**
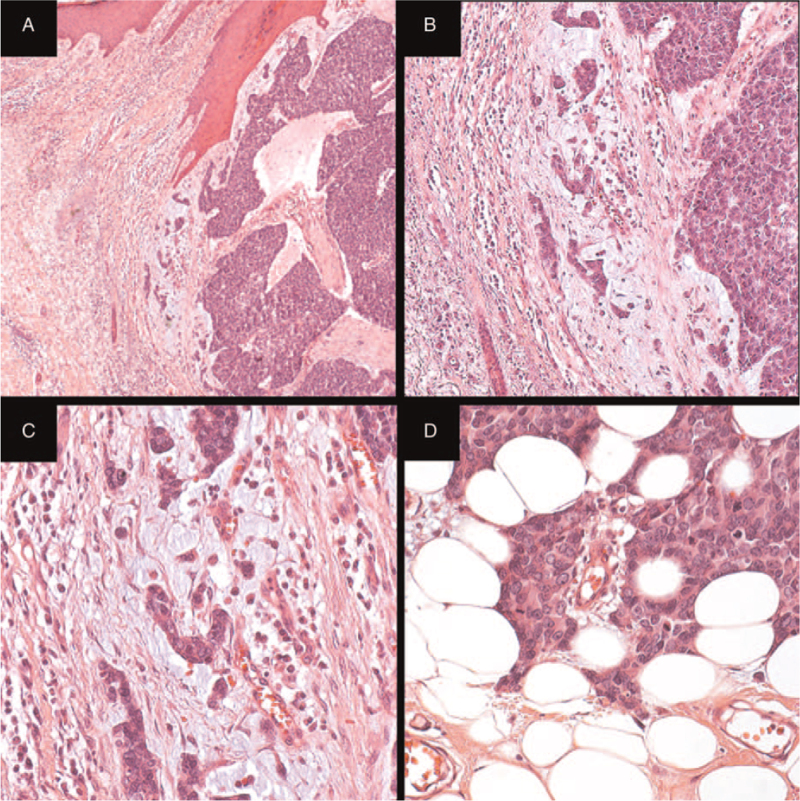
Merkel cell carcinoma: conventional optical microscopy (H&E). A (40×): transition between the tumor and adjacent normal dermis. The same field is observed in B (100×) and C (400×) where infiltrative growth pattern is evidenced. D (400×): transition between the same tumor and the underlying hypodermis. H&E = hematoxylin and eosin.

Merkel cell carcinoma (MCC) is a primary cutaneous neuroendocrine tumor induced by Merkel cell polyomavirus (MCPyV) infection and/or chronic ultraviolet radiation. The origin seems to be the Merkel cell, capable of differentiating into neuroendocrine and epithelial lineages.^[1]^ MCC is more frequent in the elderly and in patients with immunosuppression and hematologic malignancies.^[2]^ Histologically, there are 3 types of MCC: trabecular, intermediate (the most common), and small cell, but this has no clinical relevance^[2,3]^ (Fig. 1). MCC is an aggressive tumor: the 5-year survival rate ranges between 13% and 63%. The prognosis mainly depends on the stage at diagnosis. Primary tumors larger than 2 cm and/or locoregional/distant metastases are unfavorable prognostic factors. In addition, male gender, immunosuppression, infiltrative growth pattern, and lymphatic invasion are associated with reduced patient survival. Locoregional metastases are present in almost 35% of patients at diagnosis.^[4,5]^ MCCs infected with MCPyV tend to have a better prognosis, but this is debatable.^[6,7]^ The search for new therapeutic modalities, such as targeted molecular therapy, has been carried out,^[8]^ but no biomarker has been shown to be effective, either as a prognostic factor or as a therapeutic target.

It is known that cancer progression is influenced by the cross-talk between cancer cells and the extracellular matrix (ECM).^[9]^ Knowing the properties of the ECM may open the way for the identification of possible antineoplastic targets. Solid tumor-associated ECM usually contains high levels of fibrillar collagen, especially collagen types I and III, and increased collagen deposition in the tumor stroma can promote cancer progression.^[10,11]^ This is because collagen or even collagen degradation products generated by the action of metalloproteinases bind to neoplastic cell receptors and trigger multiple pathways involved in the stimulation of cell growth, motility, and inhibition of apoptosis.^[12]^ Furthermore, ECM rigidity in solid tumors is largely mediated by collagen organization. Increased enzymatic crosslinking of collagen fibers, especially by lysyl oxidases, and lysyl hydroxylases, leads to a more rigid matrix. This mechanical property can induce the epithelial–mesenchymal transition, stimulating the migratory cell behavior.^[13]^ Therefore, biochemical and biophysical cues from altered collagen fibers, associated with enlarged tumors, generally favor the progression of neoplastic cells in solid tumors.

Given the above, the aim of this study was to evaluate the collagen fibers in the stroma that accompany the MCC, using second harmonic generation (SHG) microscopy and texture analysis. In the last decade, SHG microscopy has become a powerful and widely used tool for evaluating collagen fibers in solid tumors, aiming to understand the pathological development and improve the prognostic classification.^[14–17]^ SHG microscopy is unique because it can highlight some types of fibrillar collagen in biological tissues. After acquiring the SHG image, multiple quantitative data can be performed with each image to assess collagen fibers. We aimed to study the collagen fibers in the MCC, through texture analysis, a simple, well-established and non-subjective method,^[18]^ comparing the results with histological and epidemiological data. The results of this work can be useful in the management of these patients.

## 2 Materials and methods

We included patients of both genders, of any age, with primary tumors surgically removed from any region of the skin and followed up at our Institutional Hospital. The diagnosis of MCC was based on histological examination, immunohistochemical staining for CK20 and at least two neuroendocrine markers, as well as pertinent negative epitopes, to exclude the possibility of a melanocytic, lymphoid, or metastatic nature. The following data were collected: patient sex and age, immunesuppression, primary tumor location, tumor size, and metastatic status. Patients with previously treated primary or recurrent tumors were excluded.

**Figure 2:**
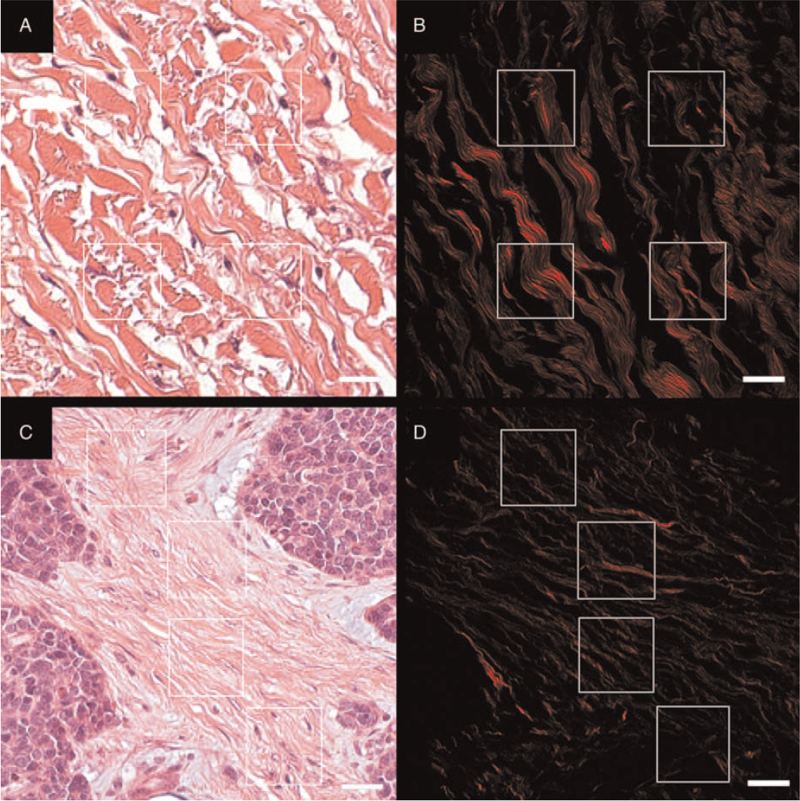
Merkel cell carcinoma: SHG microscopy. Normal dermis (A, 400×) and peritumoral stroma (C, 400×) of the same patient are observed by conventional optical microscopy (H&E). Four representative regions of interest (42.5 × 42.5 μm) were selected before SHG microscopy. Confounding factors such as skin annexes, muscle fibers, vascular structures, solar elastosis, tumoral cells, and necrotic areas were avoided. In B and D, the same fields are observed by SHG microscopy and only the collagen fibers show up in red (random color). Note that the collagen fibers are rarefied and thin in the peritumoral stroma (D). Scale bar: 20 μm. H&E = hematoxylin and eosin, SHG = second harmonic generation.

On conventional optical microscopy, the following data were collected: histological type of tumor, growth pattern and lymphatic invasion. One paraffin block was selected per case, containing tumor and adjacent normal skin, and 5-μm thick sections were stained with hematoxylin and eosin. In all cases, 2 areas were studied in the reticular dermis: normal dermis and peritumoral stroma. In each area, 2 representative ×400 fields were selected. Finally, 4 representative regions of interest (ROIs: 42.5 × 42.5 μm) were chosen in each field (Fig. 2), totaling 8 ROIs per area (normal dermis and peritumoral stroma). Skin adnexa, muscle fibers, vascular structures, solar elastosis, tumor cells, and necrotic areas were avoided.

SHG images were obtained with an inverted microscope (Axio-Observer Z1 LSM 780 - Zeiss, Oberkochen, Germany). Histological sections were excited by a Ti: Sapphire laser (Mai Tai - Spectra Physics, Santa Clara, CA) at 800 nm and the SHG signal was collected in the backward direction. SHG images (212.55 × 212.55 μm/1024 × 1024 pixels) were acquired through a large magnification (40×) oil immersion objective (EC Plan Neofluar – Zeiss, Oberkochen, Germany).

We used ImageJ (NIH, Bethesda, MD), for image analysis. The SHG images were converted into 256 gray levels. Collagen fibers texture was blindly investigated through the analysis of ROIs using first-order texture features (area fraction and mean gray value) and second-order texture features (entropy and contrast). Area fraction indicated the total area where the SHG signal was present. Mean gray value provided the mean brightness of the SHG signal, whose intensity depends on the diameter of the collagen fiber.^[19]^ Entropy values are higher when the texture of the image is less homogeneous, and various brightness levels were present. Contrast values are higher when there is a great variation in the brightness levels of the image.^[20,21]^

Statistical procedures were blindly performed using Statistical Analysis System for Windows 9.4 (SAS Institute, Cary, NC). All texture features (area fraction, mean gray level, entropy, and contrast) were compared between 2 groups. First, normal dermis was compared with peritumoral stroma, using repeated measures analysis of variance applied to rank-transformed data. Secondly, only the peritumoral stroma was evaluated, and to that end, tumors with unfavorable prognostic factors were compared with tumors with favorable prognostic factors, using generalized estimating equations. *P*-values ≤.05 were considered statistically significant.

Ethics approval: the study proceeded after approval from the Institutional Research Ethics Committee (CAAE: 69375817.6.0000.5404).

## 3 Results

**Figure 3:**
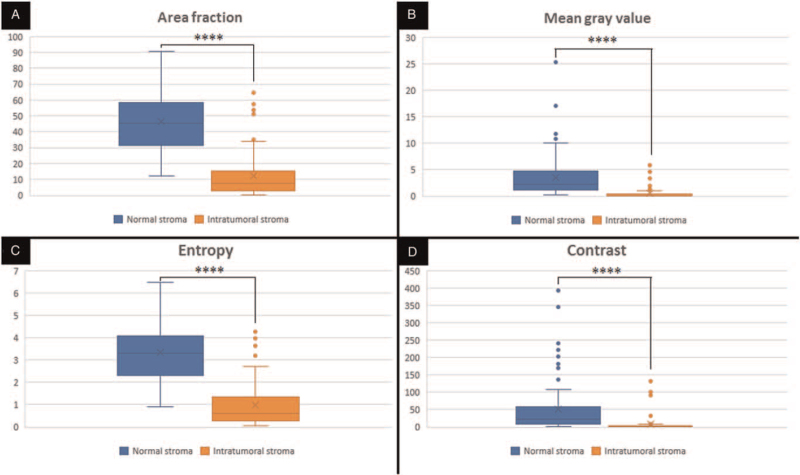
Merkel cell carcinoma: graphic representations of texture features: data extracted from the second-harmonic generation images of normal dermis (N = 11 /88 ROIs) and peritumoral stroma (N = 11 /88 ROIs). Very lower values in peritumoral stroma than the normal dermis for all the texture features [statistical test (repeated measures ANOVA applied to rank-transformed data) shows marked significant differences (^∗∗∗∗^*P* < .0001)]. ANOVA = analysis of variance, ROIs = regions of interest.

Nineteen patients were found, but only 11 met eligibility criteria. The sample was composed of 8 women (73%) and 3 men (27%), with a mean age of 72 years (range 55– 85 years). All patients were immunocompetent. Tumors were located in the head/neck region (N = 5), upper limbs (N = 2), and lower limbs (N = 4). Locoregional/distant metastases were present in 6 patients (54.5%) at diagnosis. Tumor size mean was 5.3 (range 2.2- 13.2 cm). Seven out 11 tumors (63.5%) showed infiltrative growth pattern and 4 were nodular. Lymphatic invasion was found in 8/11 (72.5%). We found significantly (*P* < .0001) lower values of area fraction (minor normal collagen density), mean gray value (minor collagen fibers diameter), entropy, and contrast (more homogeneous collagen fibers) in the peritumoral stroma, compared to normal dermis (repeated measures analysis of variance applied to rank-transformed data) (Table 1). Figure 3 illustrates these last findings. No significant differences were found when comparing the values of the density, fiber diameter, and collagen texture features, regarding factors related to an unfavorable prognosis (male gender, infiltrative growth pattern, lymphatic invasion, and locoregional/distant metastases).

**Table 1:** Comparative texture features values between normal dermis and peritumoral stroma.

Texture feature	Mean	Median	SD	Min/max	*P*-value^∗^
Area fraction (%)					
Normal dermis	46.42	45.31	17.92	12.13–90.80	<.0001
Peritumoral stroma	12.38	7.50	14.10	0.48–64.61	
Mean gray value					
Normal dermis	3.57	2.26	3.87	0.18–25.24	<.0001
Peritumoral stroma	0.54	0.14	1.07	0.01–5.82	
Entropy					
Normal dermis	3.35	3.31	1.30	0.90–6.50	<.0001
Peritumoral stroma	0.97	0.62	0.98	0.06–4.33	
Contrast					
Normal dermis	51.45	23.35	74.29	0.50–394.20	<.0001
Peritumoral stroma	7.44	0.57	22.38	0.01–131.72	

Any feature result obtained in 88 ROIs of 11 patients; area fraction: collagen density; mean gray value (collagen fibers diameter); entropy and contrast (measures heterogeneity of collagen distribution). ANOVA = analysis of variance, Min/max = minimum/maximum values, ROIs = regions of interest, SD = standard deviation.

∗ Repeated measures ANOVA applied to rank-transformed data.

## 4 Discussion

Management of MCC has been improved through a growing understanding of its pathogenesis. The study of peritumoral collagen fibers could help in this regard, as it is known that biochemical and biophysical cues from altered collagen fibers, associated with enlarged tumors, tend to favor the progression of neoplastic cells in solid tumors. Few studies have investigated the relationship between MCC and ECM macromolecules, such as lectins, proteoglycans and tenascin,^[22,23]^ but there are no studies evaluating collagen, which is the main component of ECM. We evaluated peritumoral collagen fibers using SHG microscopy and texture analysis to understand better the pathological changes that occur in MCCs. In vivo and ex vivo SHG images have been applied for the morphological study of different skin conditions.^[23–25]^ In paraffin sections, SHG signaling is not significantly affected by staining. In this study, we used paraffin sections stained with hematoxylin and eosin, as previously reported,^[26]^ to select representative ROIs containing only stroma, prior to SHG microscopy (Fig. 2A, C). After the acquisition of the SHG signal, the texture of the images was analyzed. Although conventional light microscopy shows a clear increase in collagen deposition in the peritumoral stroma (Fig. 2C), SHG microscopy shows that collagen fibers are greatly altered compared to normal dermis with fewer, thin, and more homogeneous fibers of remnant collagen still emitting SHG (Figs. 2D and 3).

Four hypotheses (singly or combined) could explain these findings. First, the collagen fibers may be enzymatically disrupted since in vitro studies show that the SHG signal is lost after collagen degeneration.^[27]^ According to this idea, fibrillar collagen types I, II, and III are hydrolyzed mainly by metalloproteinases 1, 2, 8, 9, and 13^[28]^ and it has been shown that some of these enzymes are variably expressed in MCC.^[29]^ Secondly, the collagen fibers may be enzymatically cross-linked, since in vitro studies suggest that an increase of collagen cross-linking results in a decrease of the SHG signal.^[30]^ Therefore, it would also be very interesting to carry out studies to investigate the expression profile of lysyl oxidases and lysyl hydroxylases in MCC to evaluate the relevance of cross-linking to tumor progression. Thirdly, the collagen fibers may be dissociated by edema or myxoid changes, as seen around tumor nests in Fig. 2C, since glycerol-induced fiber dissociation reduces the SHG signal in animal models.^[31]^ Interestingly, myxoid ECM of various human neoplastic tissues contains large amounts of glycosaminoglycans, proteoglycans, fibronectin and tenascin-C, which can also stimulate tumor progression.^[32]^ Fourthly, there is an increased amount of fibrillar collagen types III and/or V (or other types of fibrillar collagen) since only fibrillar collagen types I and II generate strong SHG signals.^[33]^ It is worth remembering that fibrillar collagen type III is prominent at sites of healing and repair in human skin^[34]^ and there are similarities between tumor-associated stroma and wound healing.^[35]^

Finally, we found no significant differences in any of the texture features comparing tumors with prognostic factors considered to be unfavorable with tumors with favorable prognostic factors. This suggests that MCC consistently elicits similar collagen remodeling mechanisms. In any case, all these possible alterations of collagen fibers and ECM may contribute to the aggressive behavior of MCC. Consequently, therapies using ECM macromolecules or fibroblasts (the architects of ECM remodeling) as target^[29]^ could be useful in the treatment of MCC.

The incidence of MCC is growing, but it still is a rare tumor. The low number of patients is a limitation of this work, and these are preliminary conclusions. Also, we do not assess MCPyV status in our cases. More studies are needed to determine the precise biochemical modifications of collagen in the MCC environment.

## Acknowledgments

We reviewed the content of the manuscript, followed by Ms Diane Ellis, B.A. in education. Biostatistician Cleide Aparecida Moreira Silva, Research Committee, Biostatistics Division, Medical Sciences School, Unicamp, provided statistical consultation.

## Author contributions

**Conceptualization:** Tiago Luders Laurito, Gislaine Vieira-Damiani, Carlos Lenz Cesar, Aparecida Machado de Moraes, Maria Letícia Cintra.

**Data curation:** Maria Letícia Cintra.

**Formal analysis:** Gislaine Vieira-Damiani, Vitor Bianchin Pelegati, Mariana Ozello Baratti, Hernandez Faustino de Carvalho, Carlos Lenz Cesar, Maria Letícia Cintra, Fernanda Teixeira.

**Funding acquisition:** Carlos Lenz Cesar, Maria Letícia Cintra.

**Investigation:** Tiago Luders Laurito, Flávia Thomé França.

**Methodology:** Tiago Luders Laurito, Gislaine Vieira-Damiani, Vitor Bianchin Pelegati, Mariana Ozello Baratti, Hernandez Faustino de Carvalho, Carlos Lenz Cesar.

**Resources:** Aparecida Machado de Moraes.

**Supervision:** Maria Letícia Cintra.

**Validation:** Tiago Luders Laurito, Gislaine Vieira-Damiani, Vitor Bianchin Pelegati, Mariana Ozello Baratti, Hernandez Faustino de Carvalho, Carlos Lenz Cesar, Aparecida Machado de Moraes, Maria Letícia Cintra, Fernanda Teixeira.

**Visualization:** Flávia Thomé França, Gislaine Vieira-Damiani, Vitor Bianchin Pelegati, Mariana Ozello Baratti, Hernandez Faustino de Carvalho, Carlos Lenz Cesar, Aparecida Machado de Moraes, Maria Letícia Cintra.

**Writing – original draft:** Tiago Luders Laurito.

**Writing – review & editing:** Fernanda Teixeira.

## References

[1] Narisawa Y Inoue T Nagase K. Evidence of proliferative activity in human Merkel cells: implications in the histogenesis of Merkel cell carcinoma. Arch Dermatol Res 2019;311:37–43. 30460510 10.1007/s00403-018-1877-x

[2] Chang JW Chang YY Huang YL. Merkel cell carcinoma in Taiwan: a series of 24 cases and literature review. Medicine (Baltimore) 2019;98:e17538. 31626116 10.1097/MD.0000000000017538 PMC6824798

[3] Tetzlaff MT Harms PW. Danger is only skin deep: aggressive epidermal carcinomas. An overview of the diagnosis, demographics, molecular-genetics, staging, prognostic biomarkers, and therapeutic advances in Merkel cell carcinoma. Mod Pathol 2020;33: (Suppl 1): 42–55. 31676786 10.1038/s41379-019-0394-6

[4] Coggshall K Tello TL North JP. Merkel cell carcinoma: an update and review. Pathogenesis, diagnosis and staging. J Am Acad Dermatol 2018;78:433–42. 29229574 10.1016/j.jaad.2017.12.001

[5] van Veenendaal LM van Akkooi ACJ Verhoef C. Merkel cell carcinoma: clinical outcome and prognostic factors in 351 patients. J Surg Oncol 2018;117:1768–75. 29790179 10.1002/jso.25090

[6] Uchi H. Merkel cell carcinoma: an update and immunotherapy. Front Oncol 2018;8:48. 29560342 10.3389/fonc.2018.00048 PMC5845720

[7] Nirenberg A Steinman H Dixon J. Merkel cell carcinoma update: the case for two tumours. J Eur Acad Dermatol Venereol 2020;34:1425–31. 31855292 10.1111/jdv.16158

[8] Tello TL Coggshall K Yom SS. Merkel cell carcinoma: an update and review. Current and future therapy. J Am Acad Dermatol 2018;78:445–54. 29229573 10.1016/j.jaad.2017.12.004

[9] Pickup MW Mouw JK Weaver VM. The extracellular matrix modulates the hallmarks of cancer. EMBO Rep 2014;15:1243–53. 25381661 10.15252/embr.201439246 PMC4264927

[10] Hoye AM Erler JT. Structural ECM components in the premetastatic and metastatic niche. Am J Physiol Cell Physiol 2016;310:955–67. 10.1152/ajpcell.00326.2015 27053524

[11] Fang M Yuan J Peng C Li Y. Collagen as a double-edged sword in tumor progression. Tumour Biol 2014;35:2871–82. 24338768 10.1007/s13277-013-1511-7 PMC3980040

[12] Xiong G Xu R. Function of cancer cell derived extracellular matrix in tumor progression. J Cancer Metastasis Treat 2016;2:357–64.

[13] Multhaupt HA Leitinger B Gullberg D. Extracellular matrix component signaling in cancer. Adv Drug Deliv Rev 2016;97:28–40. 26519775 10.1016/j.addr.2015.10.013

[14] Malik R Lelkes PI Cukierman E. Biomechanical and biochemical remodeling of stromal extracellular matrix in cancer. Trends Biotechnol 2015;33:230–6. 25708906 10.1016/j.tibtech.2015.01.004 PMC4380578

[15] Keikhosravi A Bredfeldt JS Sagar AK. Second-harmonic generation imaging of cancer. Methods Cell Biol 2014;123:531–46. 24974046 10.1016/B978-0-12-420138-5.00028-8

[16] Liu J. Two-photon microscopy in pre-clinical and clinical cancer research. Front Optoelectron 2015;8:141–51.

[17] Bredfeldt JS Liu Y Pehlke CA. Computational segmentation of collagen fibers from second-harmonic generation images of breast cancer. J Biomed Opt 2014;19:16007. 24407500 10.1117/1.JBO.19.1.016007 PMC3886580

[18] Ranjit S Dvornikov A Stakic M. Imaging fibrosis and separating collagens using SHG and phasor approach to fluorescence lifetime imaging. Sci Rep 2015;5:13378. 26293987 10.1038/srep13378 PMC4543938

[19] Bancelin S Aimé C Gusachenko I. Determination of collagen fibril size via absolute measurements of second-harmonic generation signals. Nat Commun 2014;16:4920. 10.1038/ncomms5920 25223385

[20] Raub CB Suresh V Krasieva T. Noninvasive assessment of collagen gel microstructure and mechanics using multiphoton microscopy. Biophys J 2007;92:2212–22. 17172303 10.1529/biophysj.106.097998 PMC1861799

[21] Mostaço-Guidolin LB Ko AC Wang F. Collagen morphology and texture analysis: from statistics to classification. Sci Rep 2013;3:2190. 23846580 10.1038/srep02190 PMC3709165

[22] Sames K Schumacher U Halata Z. Lectin and proteoglycan histochemistry of Merkel cell carcinomas. Exp Dermatol 2001;10:100–9. 11260248 10.1034/j.1600-0625.2001.010002100.x

[23] Koljonen V Jahkola T Tukiainen E. Tenascin-C in primary Merkel cell carcinoma. J Clin Pathol 2005;58:297–300. 15735164 10.1136/jcp.2004.018432 PMC1770604

[24] Monaghan MG Kroll S Brucker SY. Enabling multiphoton and second harmonic generation imaging in paraffin-embedded and histologically stained sections. Tissue Eng Part C Methods 2016;22:517–23. 27018844 10.1089/ten.tec.2016.0071 PMC4922008

[25] Chen J Lee A Zhao J. Spectroscopic characterization and microscopic imaging of extracted and in situ cutaneous collagen and elastic tissue components under two-photon excitation. Skin Res Technol 2009;15:418–26. 19832952 10.1111/j.1600-0846.2009.00381.x

[26] Vieira-Damiani G Lage D Christofoletti Daldon PE. Idiopathic atrophoderma of Pasini and Pierini: a case study of collagen and elastin texture by multiphoton microscopy. J Am Acad Dermatol 2017;77:930–7. 28389037 10.1016/j.jaad.2017.02.044

[27] Kim BM Eichler J Reiser KM. Collagen structure and nonlinear susceptibility: effects of heat, glycation, and enzymatic cleavage on second harmonic signal intensity. Lasers Surg Med 2000;27:329–35. 11074509 10.1002/1096-9101(2000)27:4<329::aid-lsm5>3.0.co;2-c

[28] Van Doren SR. Matrix metalloproteinase interactions with collagen and elastin. Matrix Biol 2015;44-46:224–31. 25599938 10.1016/j.matbio.2015.01.005 PMC4466143

[29] Lampi MC Reinhart-King CA. Targeting extracellular matrix stiffness to attenuate disease: from molecular mechanisms to clinical trials. Sci Transl Med 2018;10:0475. 10.1126/scitranslmed.aao0475 29298864

[30] Lutz V Sattler M Gallinat S. Impact of collagen crosslinking on the second harmonic generation signal and the fluorescence lifetime of collagen autofluorescence. Skin Res Technol 2012;18:168–79. 21564311 10.1111/j.1600-0846.2011.00549.x

[31] Yeh AT Choi B Nelson JS. Reversible dissociation of collagen in tissues. J Invest Dermatol 2003;121:1332–5. 14675178 10.1046/j.1523-1747.2003.12634.x

[32] Willems SM Wiweger M van Roggen JF. Running GAGs: myxoid matrix in tumor pathology revisited: what's in it for the pathologist? Virchows Arch 2010;456:181–92. 19705152 10.1007/s00428-009-0822-y PMC2828560

[33] Ranjit S Dvornikov A Stakic M. Imaging fibrosis and separating collagens using second harmonic generation and phasor approach to fluorescence lifetime imaging. Sci Rep 2015;5:13378. 26293987 10.1038/srep13378 PMC4543938

[34] Xue M Jackson CJ. Extracellular matrix reorganization during wound healing and its impact on abnormal scarring. Adv Wound Care 2015;4:119–36. 10.1089/wound.2013.0485 PMC4352699 25785236

[35] Dvorak HF. Tumors: wounds that do not heal – redux. Cancer Immunol Res 2015;3:01–11. 10.1158/2326-6066.CIR-14-0209 PMC4288010 25568067

